# Methylene blue fluorescence of the ureter during colorectal surgery

**DOI:** 10.1007/s00464-018-6219-8

**Published:** 2018-05-21

**Authors:** Thomas G. Barnes, Roel Hompes, Jacqueline Birks, Neil J. Mortensen, Oliver Jones, Ian Lindsey, Richard Guy, Bruce George, Chris Cunningham, Trevor M. Yeung

**Affiliations:** 1 Nuffield Department of Surgery, University of Oxford, Oxford, UK; 2 Department of Colorectal Surgery, Oxford University Hospitals NHS Foundation Trust, Oxford, UK; 3 Centre for Statistics in Medicine, University of Oxford, Oxford, UK; 4 Nuffield Department of Surgery, John Radcliffe Hospital, Headington, Oxford, UK

**Keywords:** Laparoscopic surgery, Fluorescence, Colorectal surgery, Ureteric injury

## Abstract

**Background:**

Iatrogenic ureteric injury is a serious complication of colorectal surgery. Incidence is estimated to be between 0.3 and 1.5%. Of all ureteric injuries, 9% occur during colorectal procedures. Ureteric stents are utilised as a method to reduce the risk of injury; however, these are not without risk and do not guarantee prevention of injury. Fluorescence is a safe and effective alternative for intraoperative ureteric localisation. This proof of principle study aims to assess the use of methylene blue to fluoresce the ureter during colorectal surgery.

**Method:**

Patients undergoing elective colorectal surgery were included in this open label, non-randomised study. Methylene blue was administered intravenously at varying doses (0.25–1 mg/kg) over 5 min, 10–15 min prior to entering ‘ureteric territory.’ Fluorescence was assessed using the PINPOINT Deep Red laparoscopic system at fixed time points by the surgeon and an independent observer.

**Results:**

42 patients received methylene blue; 2 patients were excluded from analysis. Of the 69 ureters assessed, 64 were seen under fluorescence. Of these, 14 were not visible under white light. 50 ureters were observed with both fluorescence and white light with 14 of these being seen earlier with fluorescence. In ten cases, fluorescence revealed the ureter to be in a different location than suspected.

**Conclusion:**

Fluorescence is a promising method to allow visualisation of the ureter, where it is not identified easily under standard operative conditions, thereby improving safety and reducing operative time and difficulty.

**Electronic supplementary material:**

The online version of this article (10.1007/s00464-018-6219-8) contains supplementary material, which is available to authorized users.

Iatrogenic ureteric injury is a serious and feared complication of abdominal surgery associated with significant morbidity, mortality and potential litigation to surgeons. The overall incidence is estimated to be between 0.3 and 1.5% [1–4] with more than half attributed to gynaecological procedures [3, 5, 6]. 9% of all ureteric injuries occur during colorectal resections [7] and in colorectal procedures; particularly, the lower third of the left ureter is at risk in rectal cancer cases [4]. Early detection of the ureter not only avoids inadvertent injury but also ensures that the correct plane is being dissected and thus other structures including the gonadal vessels and autonomic nerves are protected [8].

Ureteric stents have been employed as a method of facilitating ureteric identification. However, stent insertion is associated with increased operating time [9–11], overall procedure cost, increased length of stay [11], radiation exposure and potential urinary tract complications including infection, urinary retention, haematuria and perforation [9, 10]. An alternative that is quick, safe and can be used at any point in a procedure would prove useful. Fluorescence has proven to be a promising method of highlighting ureters providing real-time visualisation of their location. Intravenous methylene blue was successfully first used in humans in 2013 demonstrating its feasibility for open pelvic surgery [12]. Two small studies explored the use of methylene blue fluorescence in laparoscopic surgery [13, 14]. Methylene blue proved to be useful in eight patients using an in-house manufactured laparoscopic system, highlighting ureters without prior surgical exposure in patients undergoing open and laparoscopic colorectal surgery. In a later study, Al-Taher et al. [14] used low-dose methylene blue in ten patients, only visualising the ureters in half of their patients after the ureters had already been located under white light.

This study further assesses the feasibility of methylene blue fluorescence in the ureter in a greater number and wider range of colorectal procedures with an aim to determine the optimum dosing and timing of administration.

## Materials and methods

The study was approved by the Health Research Authority (HRA), the East Midlands—Derby Research Ethics Committee (ref: 14/EM/1107) and Oxford University Hospitals research and development department. The study was registered on clinicaltrials.gov (NCT03177070).

Patients were approached sequentially (according to availability of the research team) for inclusion in the study if they were undergoing laparoscopic or open elective colorectal surgery and were over 18 years of age. Patients with known allergies to methylene blue, the presence of risk factors for serotonin syndrome (e.g. taking a serotonin reuptake inhibitor, previous history of serotonin syndrome or GP6D deficiency) or significant renal or hepatic impairment were ineligible to enter the study. Female patients that were pregnant and/or breast feeding were also not eligible.

### Administration of methylene blue

Standard anaesthetic and surgical procedures were carried out for all operations. Methylene blue (5 mg/ml) was administered intravenously over 5 min, 10–15 min prior to the surgeon entering ureteric territory followed by a saline flush. Dosing cohorts were based on previous literature [13–15] and included 0.25, 0.5, 0.75 and 1 mg/kg.

### Fluorescence assessment

The PINPOINT Deep Red (DR) laparoscopic system (NOVODAQ, USA) was the NIR fluorescence enabled system used for the study. This system gives surgeons the ability to visualise in a white light only and convert to a fluorescence mode which displays a white light image, a fluorescent only black and white image (SPY mode) and an overlay of fluorescence (green) to the white light image.

Once methylene blue was administered, visualisation of the ureter(s) under fluorescence was attempted at time 0, 5, 10 min and every 10 min thereafter where it was safe an appropriate to do so for up to 100 min. Upon entering the region of the ureter an assessment was made initially under white light, followed by fluorescence. Visualisation under both white light or fluorescence was deemed either ‘visible’ or ‘not visible’. Visibility was defined as the surgeon being 100% certain that the ureter is localised. No additional dissection to explore the ureter(s) was performed outside of the standard operative procedure to identify fluorescence. Discretion was given to the operating surgeon as to whether techniques were employed to identify a ureter not identified with white light but with fluorescence. According to the procedure being undertaken, the ureters were classified as being either relevant or irrelevant to the procedure. Examples of relevant ureters included: left ureter in an anterior resection, both ureters during subtotal colectomy, right ureter during a right hemicolectomy. Irrelevant ureters were those that would not be routinely looked for, but were observed under fluorescence for the purposes of this study. Documentation was made as to whether the ureter was visible with white light alone, fluorescence alone, both fluorescence and white light or not visible at all by the operating surgeon (primary observer) and an independent (secondary) observer. Where possible, a final assessment of the ureter(s) was made at the end of the procedure to observe whether fluorescence was still present. The order in which ureters were assessed were as follows: assessment under white light only, assessment under fluorescence (including overlay and pure fluorescence modes).

Post-operatively, recorded footage was analysed and signal-to-background ratio of ureteric fluorescence at the time intervals was calculated using ImageJ [16] utilising the SPY mode image only. A region of interest was manually drawn around the visualised fluorescence in the ureter and the same sized region placed over a random background area. Brightness levels were calculated in each region (an average of intensity of each pixel within the region) and the signal-to-background ratio calculated [17].

Fluorescence was deemed ‘useful to the case’ where the surgeon used fluorescence mode without prompting outside of the fixed time point.

Adverse events were collected prospectively for 30-day post-operatively.

### Statistical analysis

Descriptive statistics were used to present the majority of data arising from the study. Comparison of means across all times points between dosing cohorts from signal-to-background ratio data was assessed using a one-way analysis of variance. Ureteric visualisation with methylene blue or white light was analysed using generalised linear mixed models. Random coefficient models were fitted to allow for differences between the change over time between patients, and the change over time was modelled using a second order regression equation. Baseline characteristics including dosing level were included as fixed effects to investigate whether these may explain variation between patients. Intraobserver agreement was assessed using the kappa statistic.

Signal-to-background ratio over time was assumed to have a normal distribution, and linear mixed models were used to analyse this change over time modelled using a second order regression equation. A random coefficient model and random intercept was fitted which allowed for differences in the change over time between patients. Where there was no superiority of either method to fit the data, the random coefficient model was utilised for simplicity. To model the correlation over time within patients, the unstructured covariance matrix was used. Baseline characteristics including dosing level were included as fixed effects to investigate whether these may explain any variation between patients.

## Results

**Fig. 1:**
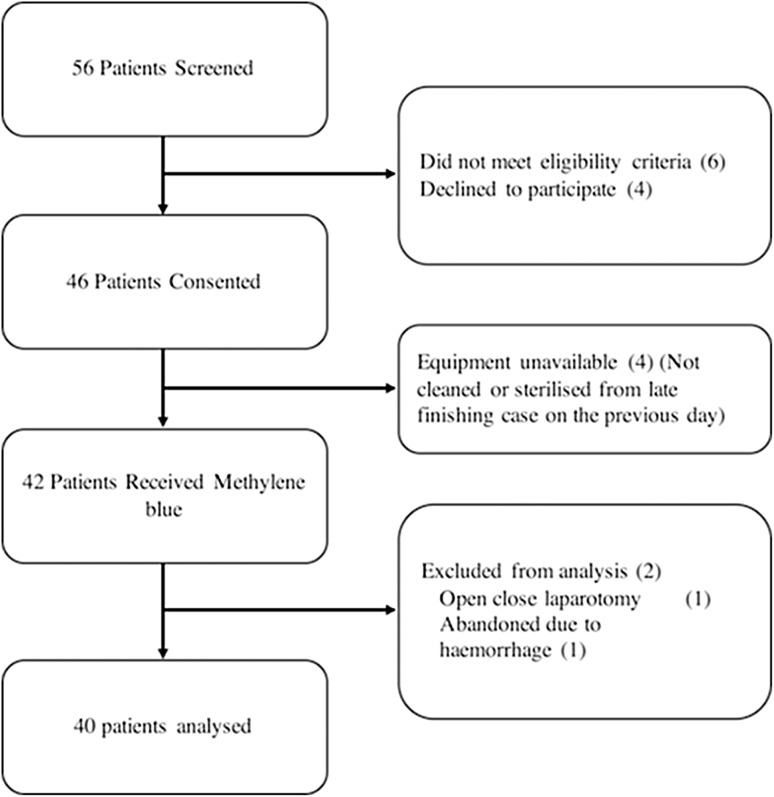
Flow diagram outlining patient recruitment

From August 2016 to April 2017, 56 patients were screened to participate in the study. 40 patients assessing 69 ureters (6 in open cases) were included in the final analysis (Fig. 1). Table 1 outlines the patient characteristics. The number of patients in each dosing cohort was as follows: 4 for 0.25 mg/kg; 11 for 0.5 mg/kg; 12 for 0.75 mg/kg; and 13 for 1 mg/kg.

**Table 1:** Procedure and indication for included patients

Patient characteristic	*N*
Median age (range)	66 (39–91)
Median BMI (range)	27 (13–39)
Gender, *N* (%)	
Male	21 (52.5)
Female	19 (47.5)
Approach, *N* (%)	
Open	4 (10)
Laparoscopic	36 (90)
Procedure and indication, *N*	
Anterior resection—cancer	19
Anterior resection—diverticular disease	3
Anterior resection—endometriosis	1
Right hemicolectomy—cancer	6
APER—cancer	2
Subtotal colectomy—cancer	1
Subtotal colectomy—UC	1
Hartmann’s—cancer	1
Hartmanns—diverticular disease (colovaginal fistula)	1
Re-do anterior resection—anterior resection syndrome	1
Re-do proctocolectomy—recurrent cancer	1
Right hemicolectomy, anterior resection and bilateral oophorectomy—cancer	1
Panproctocolectomy—Crohn’s	1
Reversal of hartmann’s	1

*BMI* body mass index, *APER* abdomino-perineal excision of the rectum, *UC* ulcerative colitis

The kappa coefficient of agreement between the observer and the surgeon was 1.00. In 40 patients, 69 ureters were assessed for fluorescence (43 relevant, 26 irrelevant). Of the 11 ureters not assessed, the majority were irrelevant to the procedure: left ureters in a right hemicolectomy (*n* = 6); right ureters during a left sided resection (*n* = 4). One relevant ureter was not assessed and this was the right ureter during a subtotal colectomy.

**Fig. 2:**
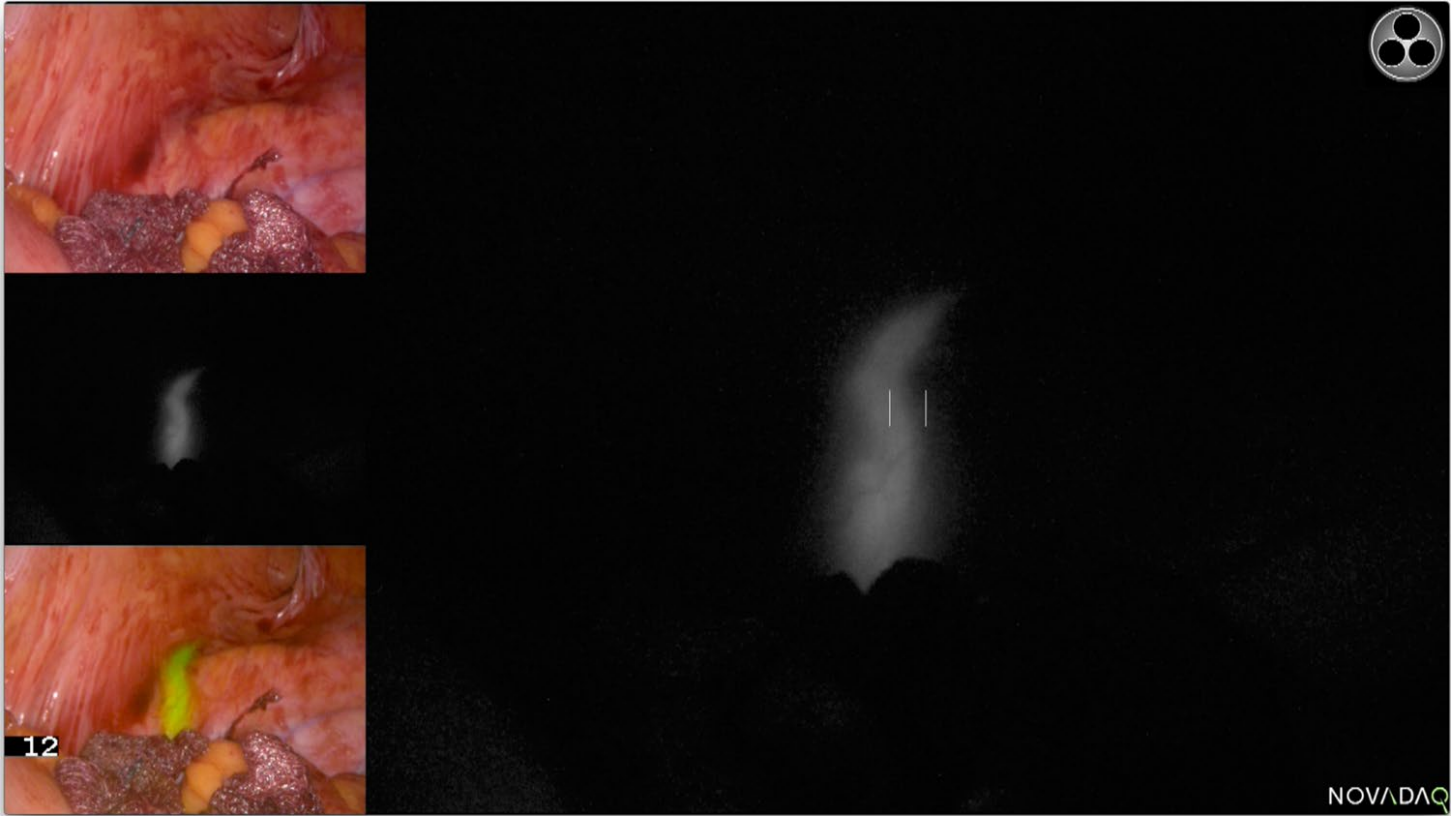
Example of fluorescence seen in the right ureter during an anterior resection prior to peritoneal incision for medial to lateral mobilisation. The three images on the left depict a white light image at the top, fluorescence only (SPY mode) in the middle and overlay at the bottom. The main display is now demonstrating the SPY mode. Note that the ureter is well covered by peritoneum and not clearly visible in the white light image (top left)

**Fig. 3:**
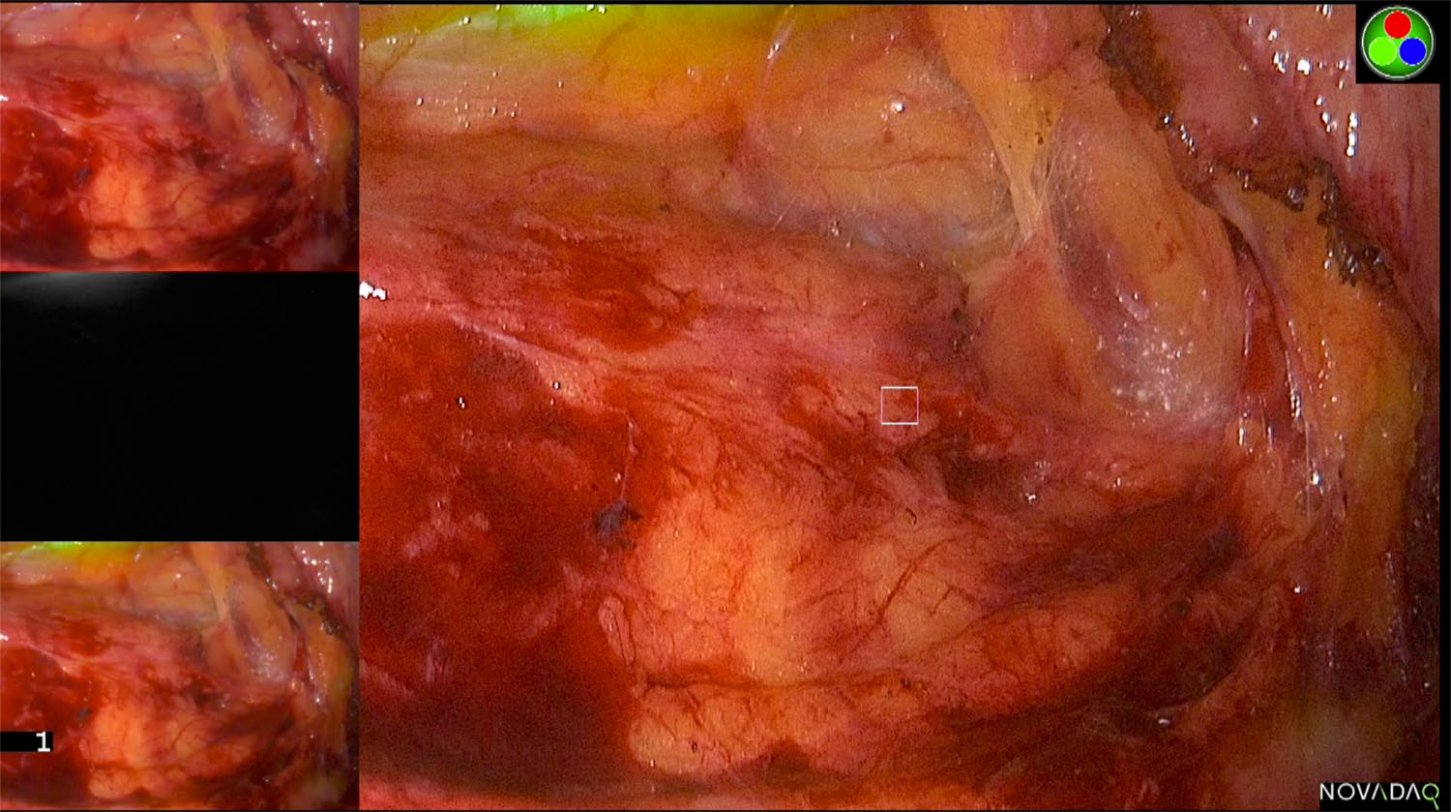
Image demonstrating attempted ureteric identification during anterior resection. The surgeon believes the ureter is where the white square is on the main display. However, with the fluorescence, the ureter is demonstrated to be more lateral than though (top of screen)

**Fig. 4:**
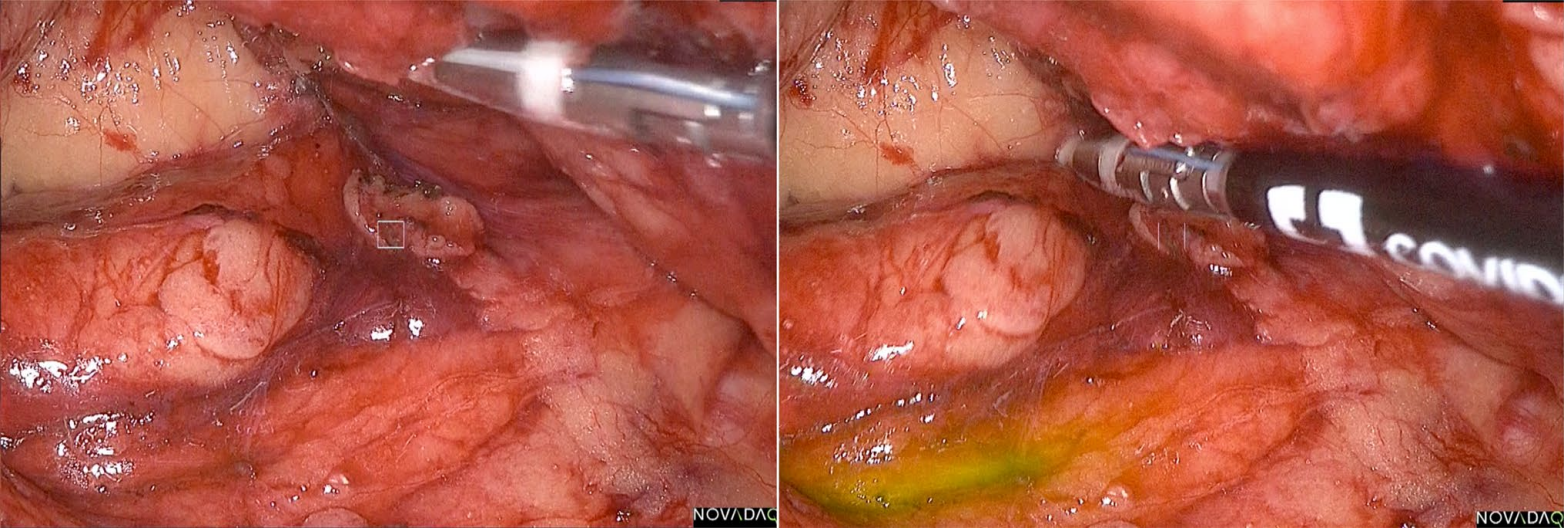
Anterior resection, left ureter. Image on the left is without fluorescence and on the right shows fluorescence mode active with overlay. Note that the ureter is not visible without fluorescence

The majority of ureters (93%) were seen under fluorescence. 14 (20%) of all ureters were clearly visible only under fluorescence and not seen with white light or any further dissection throughout the entire case. A further 14 ureters were seen first with fluorescence and subsequently white light (median 30 min later) and 6 were seen with white light and later fluorescence (10 min later). 30 ureters (43%) were deemed ‘easy ureters’ where the ureter was immediately visible under both modalities upon entering the plane. Of the remaining ureters, 4 were not seen with either modality and 1 was seen under white light but not fluorescence. Table 2 outlines these results broken down by relevance of the ureter to the procedure. Example images are shown in Figs. 2, 3 and 4.

**Table 2:** Visualisation results for relevant and irrelevant ureters under each modality

Number	Relevant ureters		Irrelevant ureters	
	Laparoscopic	Open	Laparoscopic	Open
Total assessed	39	4	24	2
Seen with fluorescence only, *N*	8	1	5	0
Seen with white light only, *N*	0	0	0	1
Not seen with either modality, *N*	1	0	3	1
Seen with both modalities				
Methylene blue first, *N*	10	2	2	0
White light first, *N*	5	0	1	0
Both first, *N*	15	1	14	0

**Fig. 5:**
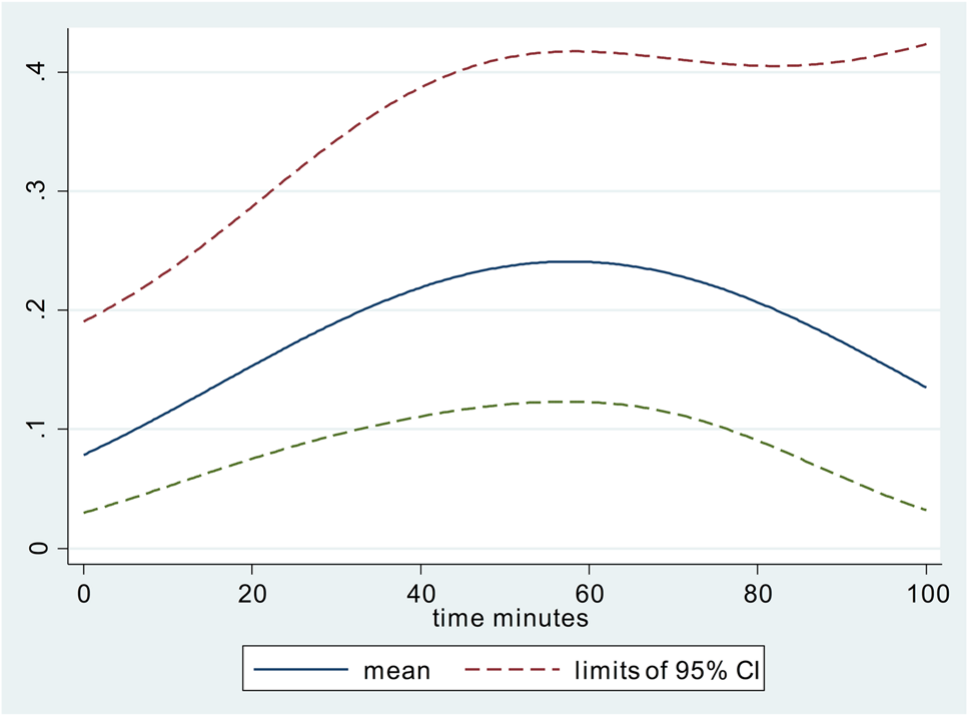
Mean probability of methylene blue being better than white light in seeing the ureter over time. Dotted line represents upper and lower 95% confidence intervals

Overall, one is more likely to see the ureter with fluorescence than with white light (OR 4.5176, 95% CI 3.33–6.13, *p* < 0.0001) and this varies over time (Fig. 5). The results of the analysis of the binary variable, the visibility of the ureter with methylene blue, show that the probability increases over time until a maximum was reached at 58 min from baseline (*p* = 97%). The following covariates were included in the model as fixed effects, age, sex, BMI, previous surgery, type of surgery, and dose. The only significant factor was sex, with the odds ratio of 3.3 (95% CI 1.0, 10.5, *p* = 0.05) for female compared with male for visibility withy methylene blue. The estimated mean probability (95% CI) of seeing the ureter with methylene blue over time is shown in Supplementary Fig. 1.

### Subjective assessment

Fluorescence was deemed ‘useful’ in 13 cases (none of these were open cases) where in 10, fluorescence revealed the ureter to be in a location different to where the surgeon predicted (Fig. 3) and in 2, the operative plan was altered. One case was during an anterior resection for severe diverticular disease with the colon adhered to the retroperitoneum. After dividing the inferior mesenteric artery (IMA), there was concern that the ureter may have been injured. Under fluorescence, the ureter could be seen well away from the area of dissection, preventing further dissection to identify the ureter. The second case was an obese patient during an anterior resection where the left ureter could not be seen with white light but was clearly seen with fluorescence (Supplementary Video 1). Fluorescence was not deemed useful in any of the open cases.

### Adverse events

Methylene blue caused an artificial drop in oxygen saturations measured via pulse oximeter in all patients and recovered moments after administration. All patients had green-stained urine post-operatively which cleared during their recovery. There were no adverse events after administration of methylene blue.

### Dosing and timing

The rate of change of the signal-to-background ratio for each of the four dosing cohorts was analysed by fitting a random intercept model including the dose group as a continuous variable replacing each level by the dose of methylene blue administered. The following covariates were added to the model as fixed effects: dose of methylene blue, age, sex, BMI, type of surgery and previous surgery. The only significant factor was sex.

Signal-to-background ratio declines over time with all doses. Using a random intercept regression model, it was confirmed that signal-to-background ratio declines most rapidly over time with 0.25 mg/kg (slope = − 0.046 (95% CI − 0.073, − 0.018) *p* = 0.001) and hardly at all with 1 mg/kg (slope = − 0.006 (95% CI − 0.023, 0.011) *p* = 0.5) with the rate of decline decreasing as the dose increased (Supplementary Table 1). Significant difference was seen between mean signal-to-background ratio over all time points between doses (*p* = 0.0005). The highest mean signal-to-background ratio was 0.75 mg/kg (mean = 5.29, SD = 2.72, 95% CI 4.84–5.75), and the lowest was observed at 1 mg/kg (mean = 3.66, SD = 1.89, 95% CI 3.37–3.39).

The signal-to-background ratio at baseline (time = 0) and the rate of change of the ratio over time depended on dose. At baseline, the lower the dose the higher the ratio, at 43 min the ratio was equal for all doses, and at 100 min, the higher the dose the higher the ratio.

The earliest fluorescence was observed in the ureter was during injection of methylene blue (time zero) and the longest observed signal time was 2 h and 5 min post injection.

## Discussion

This study demonstrates that image-guided surgery with real-time fluorescent visualisation of the ureters is a simple, effective and safe technique. The ideal time to administer methylene blue is likely to be around 10–15 min prior to requiring ureteric visualisation. Whilst 1 mg/kg had the slowest rate of decline in signal-to-background ratio, 0.75 mg/kg had the highest signal-to-background ratio compared with all other dosing cohorts and is therefore likely to be the optimum dose. The effect of volume was not assessed as the concentration was the same at 5 mg/ml in all patients and therefore volume completely relates to weight and dosing cohort. In the UK, methylene blue is licensed for weight based prescribing and therefore it was not feasible to assess fixed volumes in this study.

In this study, only one failed case of a relevant ureter not being visible under fluorescence was observed. This was the right ureter during a limited right hemicolectomy where the ureter could not be seen under white light either. There were a number of occasions where the ureter was not seen at all with white light for the whole procedure but was clearly seen under fluorescence (20%). Whilst this seems a high rate of non-visualisation with white light it is due to the fact that in this study, discretion was given to the surgeon as to whether additional dissection was to be performed to positively identify the ureter under white light. In these cases, the ureteric fluorescence was clear enough to assure the surgeon that the ureter was positively identified and therefore no additional dissection required to identify the ureter under white light. A ‘bolus’ of fluorescence in a tubal pattern was the clearest indicator that the ureter was fluorescing.

The strongest fluorescent signal in the ureter was observed during vermiculation when a bolus of methylene blue would be seen. There were a number of instances where static fluorescence was noticed; however, the intensity always seemed to be lower. This may sometimes be confused with low-level signal in the vasculature or other tissue (e.g. psoas muscle). In the experience from this study, a bolus of strong fluorescent signal during vermiculation proved to be the most reliable way of identifying the location of ureters under fluorescence.

Success of this technique was also observed in open cases using the laparoscope to assess the fluorescence although the numbers were small. Whilst we still observed ureteric fluorescence, there are some disadvantages with using this technique in open surgery including the necessity for darkness during assessment, the excitation light being in the far red making operative field visualisation difficult and the ergonomics of using a laparoscope in an open abdomen. These draw backs may be barriers in allowing surgeons to operate under fluorescence guidance in the open abdomen; however, wide field cameras are being developed that negate the need for complete darkness in the operative field to observe fluorescence.

Prior to fluorescent technology, ureteric stents have long been the modality by which surgeons identify and protect the ureter. These however come with additional cost and time constraints, and, as they rely on tactile feedback, are less suitable in laparoscopic procedures. Lighted stents have been explored to overcome the necessity to rely on tactile feedback of ureteric position; however, the only outcome assessed has been ureteric injury for which the studies are clearly underpowered [9, 18, 19]. No assessment was made on whether lighted stents assist surgeons in identifying the ureter sooner. Fluorescent technology is quick, safe and can be used at any point during the procedure.

Our study included re-operative, inflammatory bowel disease, fistulating diverticular disease and endometriosis cases where stents may have been considered by some surgeons. The success of methylene blue in these cases demonstrates that this technology may in future negate the need for ureteric stents during colorectal surgery.

Methylene blue is a dye that has long been used in humans with a good safety profile. Only recently has the discovery of its NIR fluorescent properties been evident at micromolar concentrations [15].. However, its excitation (668 nm) and emission (688 nm) are still in the visible light spectrum (400–700 nm) and therefore there may be some autofluorescence which can confuse the displayed image. The visualisation of methylene blue fluorescence may also limited by a reduced renal function (an exclusion criteria in our study) and some patients may metabolise methylene blue to the non-fluorescing leucomethylene blue although it is not known what influences this [20].

The cost of methylene blue per patient is around £70 (~ $90), although it is not yet approved for this use by the FDA or MHRA. Whilst the PINPOINT DR system is not currently commercially available, fluorescence enabled laparoscopic systems tuned to ICG cost from £110,000 to £150,000. Dual wavelength systems incorporating compatibility with methylene blue are still under evaluation.

Methylene blue has a low quantum yield (ratio of the number of photons emitted to the number of photons absorbed) [15] meaning that it is not a very efficient fluorophore with a low ‘brightness’. Other ‘brighter’ fluorophores further in the NIR spectrum may provide increased fluorescence using a similar technique. Indocyanine green (ICG) is a fluorophore that is higher in the NIR spectrum than methylene blue and has a higher depth of penetration through tissue as well as a good safety profile. It is a widely used fluorescent dye in numerous surgical fields and would be an ideal fluorophore for ureteric fluorescence; however, as it binds to plasma proteins it is thus excreted via the hepatobiliary system [21], and therefore does not reach the ureter when injected intravenously. Direct ureteral injection with ICG is possible and has been reported in a number of patients [22–24] but still requires instrumentation of the bladder and ureteric orifice. Fluorophores that are renally excreted with similar emission wavelengths to ICG are currently in development.

### Study limitations

This proof of principle study is limited by a small heterogenous cohort of patients. There is likely an interaction between interpretation of a fluorescent and white light image for ureteric localisation therefore introducing observation and measurement bias. Furthermore, this study was conducted in a specialist colorectal centre where fluorescence technology is in use for most cases. Our use of a binary subjective outcome limited our results to only 100% certainty of the ureter being visible. Although there are currently no scoring systems in the literature for subjective fluorescence assessment, such a tool may be useful in future studies. Finally, assessment of the learning curve with this technology was not under the remit of this proof of principle study but should be considered in future trials.

This proof of principle study has provided the foundations for further, larger, clinical trials evaluating this technology. Due to the low incidence of ureteric injury, to power a randomised trial using this as a primary outcome measure would require a very large patient group. It may be useful to explore whether ureteric fluorescence decreases operating time at specific points during surgery (e.g. from incision of peritoneum for medial to lateral dissection to ligation of the IMA during anterior resections), or whether the use of fluorescence changes surgical management intra-operatively.

This study has outlined a safe and easy technique for successfully fluorescing the ureter intra-operatively using methylene blue. As well as providing reassurance for the surgeon, it may be an important technology to make surgery safer.

## Electronic supplementary material

Below is the link to the electronic supplementary material.

Supplementary Figure 1 – Probability of seeing the ureter with methylene blue fluorescence over time. Blue line indicates the mean and dotted lines indicate the 95% confidence intervals. (TIFF 95503 KB)

Supplementary Table 1 – Baseline and slope for signal to background ratio for the 4 dose levels of methylene blue (males only). Females show a lower signal to background ratio for the intercepts (− 1.445 (95% CI − 2.28 – 0.61) p = 0.001) but the slopes are identical to the values for males. (DOCX 38 KB)

Supplementary Video 1 – 62 year old male undergoing laparoscopic anterior resection with a BMI of 35. Difficulty visualising the ureter with white light due to thickness of pre-peritoneal fat. Fluorescence clearly identifies the ureter preventing further dissection. (MP4 20524 KB)

Supplementary Video 2 – 56 year old male undergoing a re-do laparoscopic anterior resection for anterior resection syndrome. Demonstration of real-time ureteric fluorescence during dissection of the colon adhered to the left pelvic brim. (MP4 15564 KB)
