# Supplementary material for: Methylene blue fluorescence of the ureter during colorectal surgery
Source: Surg Endosc. 2018 May 21;32(9):4036–43. doi: 10.1007/s00464-018-6219-8 (PMC6096537; doi:10.1007/s00464-018-6219-8)
Supplement: Supplementary file 2 — Supplementary Table 1 – Baseline and slope for signal to background ratio for the 4 dose levels of methylene blue (males only). Females show a lower signal to background ratio for the intercepts (− 1.445 (95% CI − 2.28 – 0.61) p = 0.001) but the slopes are identical to the values for males. (DOCX 38 KB) [file 464_2018_6219_MOESM2_ESM.docx]

| Dose of methylene blue | Baseline intercept 95% CI | Slope units/minute 95% CI |
| --- | --- | --- |
| 0.25 mg/kg | 7.35 (5.99, 8.72) | -0.046 (-0.073, -0.018) p=0.001 |
| 0.50 mg/kg | 6.59 (5.68, 7.50) | -0.033 (-0.049, -0.016) p<0.0001 |
| 0.75 mg/kg | 5.83 (5.10, 6.55) | -0.019 (-0.030, -0.008) p=0.001 |
| 1.00 mg/kg | 5.06 (4.09, 6.04) | -0.006(-0.023, 0.011) p=0.5 |
